# Effects of a mixture of chloromethylisothiazolinone and methylisothiazolinone on peripheral airway dysfunction in children

**DOI:** 10.1371/journal.pone.0176083

**Published:** 2017-04-28

**Authors:** Hyun-Ju Cho, Dong-Uk Park, Jisun Yoon, Eun Lee, Song-I Yang, Young-Ho Kim, So-Yeon Lee, Soo-Jong Hong

**Affiliations:** 1 Department of Pediatrics, Childhood Asthma Atopy Center, Environmental Health Center, Asan Medical Center, University of Ulsan College of Medicine, Seoul, Korea; 2 Department of Environmental Health, Korea National Open University, Seoul, Korea; 3 Department of Pediatrics, Chonnam National University Hospital, Gwangju, Korea; 4 Department of Pediatrics, Hallym University Sacred Heart Hospital, Hallym University College of Medicine, Anyang, Korea; 5 Department of Pediatrics, Gyeongsang National University Changwon Hospital, Changwon, Korea; National Yang-Ming University, TAIWAN

## Abstract

**Background:**

Children who were only exposed to a mixture of chloromethylisothiazolinone (CMIT) and methylisothiazolinone (MIT) as humidifier disinfectant (HD) components were evaluated for humidifier disinfectant-associated lung injury (HDLI) from 2012. This study was to evaluate the pulmonary function using, impulse oscillometry (IOS) for children exposed to a mixture of CMIT/MIT from HD.

**Methods:**

Twenty-four children who were only exposed to a mixture of CMIT/MIT, with no previous underlying disease, were assessed by IOS. Diagnostic criteria for HDLI were categorized as definite, probable, possible, or unlikely. Home visits and administration of a standardized questionnaire were arranged to assess exposure characteristics.

**Results:**

Definite and probable cases showed higher airborne disinfectant exposure intensity during sleep (32.4 ± 8.7 μg/m^3^) and younger age at initial exposure (3.5 ± 3.3 months) compared with unlikely cases (17.3 ± 11.0 μg/m^3^, p = 0.026; 22.5 ± 26.2 months, p = 0.039, respectively). Reactance at 5 Hz was significantly more negative in those with high-density exposure during sleep (mean, -0.463 kPa/L/s vs. low density, -0.296, p = 0.001). The reactance area was also higher with high-density exposure during sleep (mean, 3.240 kPa/L vs. low density, 1.922, p = 0.039). The mean bronchodilator response with high-density exposure was within the normal range for reactance.

**Conclusions:**

Significant peripheral airway dysfunction were found in children with high levels of inhalation exposure to a mixture of CMIT/MIT during sleep. Strict regulation of a mixture of CMIT/MIT exposure were associated with positive effects on lung function of children.

## Introduction

Isothiazolinones are broad-spectrum biocides that are widely used in many water-based consumer products, including personal hygiene products, perfume, wall paint, and deodorants to prevent growth of microorganisms [1, 2]. Their sterilizing properties are also used in humidifier disinfectant (HD) and air conditioning systems [3]. Since the 1980s, the most frequently used isothiazolinones have been 5-chloro-2-methyl-4-isothiazolin-3-one (CMIT) and 2-methyl-4-isothiazolin-3-one (MIT), usually in a 3:1 mixture [1] and the major chemical disinfectants contained in HD were also a mixture of CMIT/MIT and other chemical compounds including polyhexamethylene guanidine phosphate (PHMG), and oligo(2-(2-ethoxy) ethoxyethyl guanidinium (PGH).

A mixture of CMIT/MIT is well known to cause allergic dermatitis responses via contact with the air although rare in the respiratory disease [4–8]. However, the Korea Centers for Disease Control and Prevention (KCDC) under the Ministry of Health and Welfare declared HD as a cause of lung injury and recalled HD products in November 2011. To identify humidifier disinfectant-associated lung injury (HDLI) in Korean children, we first published a case-series study [9, 10] and then a case-control study [11]. We also confirmed that the nationwide suspension of HD sales in 2011 decreased HDLI incidence through a nationwide, real-time reporting system for the entire Korean pediatric population [12]. As in children, 17 adult cases of HDLI were reported [13], and a case-control study showed that only HD use was a statistically significant factor for interstitial lung disease (ILD) when the cases were compared with each of the control subgroups [14].

In animal studies performed by the KCDC, inhalation of PHMG and PGH revealed histopathological readings identical to those of patients with HDLI in November 2011 [10, 15, 16]. However, a mixture of CMIT/MIT has not been proven in a laboratory experiment to be directly related to HDLI despite the presence of patients diagnosed with HDLI, unlike PHMG and PGH. As a result, these individuals are facing difficulties in receiving acknowledgment of the health impact of these compounds and are calling for a comprehensive pulmonary evaluation of toxicity related to a mixture of CMIT/MIT.

Inhalational injury was known to be associated with pathologic changes in the distal airways including respiratory bronchioles and alveolar ducts [17, 18], which may not be apparent on chest radiographs [19]. Moreover, previous animal studies also investigated the effect of inhalation of a mixture of CMIT/MIT, which has been shown to cause pulmonary toxicity in rats [4, 20]. Therefore, it is important to determine the potential functional loss that can be caused by inhalation exposure to a mixture of CMIT/MIT. Sixty percent of those diagnosed with HDLI were under 8 years of age at the visit for pulmonary function. Impulse oscillometry (IOS) is known to be useful in preschool children as it can easily measure pulmonary function with passive cooperation [21]). Hence, we evaluated pulmonary function using IOS in children exposed to a mixture of CMIT/MIT.

## Materials and methods

### Subjects and design

The Korean Government completed the third round of investigation (first: July 2013 through April 2014; second: July 2014 and April 2015; and third: September 2015 through August 2016) on the association between HDLI and the use of HD by collecting information on individuals who presumed their condition was related to the use of HD and determined whether these registered cases were indeed associated with the use of HD.

Of the 360 total children who were registered in the first, second, and third rounds of investigations, 29 children were exposed solely to a mixture of CMIT/MIT, excluding 196 exposed solely to PHMG, 21 exposed solely to PGH, 105 exposed to multiple compounds, and three exposed to Sodium Dichloro-S-Triazinetrione (SDT).

Among them, five children who had underlying histories were excluded including two preterm births, one with Roland syndrome, one with a history of myocarditis, and one with congenital heart disease.

Therefore, 24 children with no previous underlying disease who were exposed to only a mixture of CMIT/MIT were chosen as subjects for the IOS investigation. Height (cm), weight (kg), and body mass index (BMI; kg/m^2^) were measured at the visit for IOS measurement. The study protocol was approved by the Institutional Review Board of the Asan Medical Center (approval number: 2015–0510), which waived the requirement for informed consent because the study was retrospective in design and all patient records were anonymized and de-identified prior to analysis. This research was conducted in accordance with the principles of the Declaration of Helsinki.

### Terminology

Each subject’s age at initial HD exposure was recorded in months. Exposure from the fetal period was expressed as 0 months, and exposure only during the fetal period (n = 1) was excluded. Fetal exposure means an exposure that began during the fetal period and continued to after birth, or exposure only during the fetal period. Intensive use means use regardless of the season for less than 2 years, and intermittent use means sporadic use only during the winter season for over 2 years. The question used on the environmental investigation questionnaire was total use (months).

### Diagnostic criteria

The investigation committee evaluates the degree of damage by compiling results from individual tests such as environmental exposure, histopathology, radiology, and clinical tests.

The classifications for being identified as having lung disease caused by HD and the definitions of the respective categories of judgement are definite, probable, possible, and unlikely and are discussed in our previous paper [22, 23] The final decision regarding the identification of lung disease due to HD is made after a final review by the Environment and Health Committee (deputy chairman of the Ministry of Environment).

Among the total of 360 registered children (169, 66, and 125 individuals from the first-, second-, and third-round investigations, respectively), 161 individuals (44.7%) were classified as “definite” or “probable.” The rest were classified as “possible,” “unlikely,” or “indeterminate” because of a lack of data.

### Assessment of the exposure index to humidifier disinfectant

The methods used for investigating use characteristics of HD based on personal interviews and home investigations have been described elsewhere [16, 24, 25]. The exposure assessment team was blinded to the study subjects’ clinically diagnosed lung disease information. Three trained environmental health scientists conducted both a home investigation and personal interview related to the use of HD and demographic characteristics. We asked study participants, or in the case of children, their parents or guardians, to complete detailed questionnaires collecting information related to HD use. To aid in recall, participants were shown photographic examples of all the HD products that had been marketed in South Korea.

The HD use characteristics used for this study were extracted as follows:

1. Average hours sleeping in a room with an operating humidifier treated with disinfectant
2. Average distance (in meters) of the bed from the humidifier (< 0.5 m, 0.5 ≤ distance < 1 m, 1 ≤ distance < 2 m, 2 ≤ distance)
3. Total months that HD was used
4. Direction that the humidifier dispersed (forward vs. diagonal)
5. The presence of intense HD use (yes vs. no)
6. Airborne HD level (C, μg/m^3^) was estimated based on the level of a mixture of CMIT/MIT, the total HD volume (mL), hours of use per day (h), room size in which the HD was used (m^3^), and assumed ventilation rate. The levels of mixtures of CMIT/MIT in HD brands reported by the KCDC were used to estimate the airborne generation rate (μg/h). Air change per hour (ACH) in the room was assumed to be 0.5. Ventilation rate (Q, m^3^/h) was calculated based on the room size and an ACH of 0.5. Finally, airborne HD level was estimated by C and Q.

### Pulmonary function test: Impulse oscillometry (IOS)

IOS was performed using the Master Screen Spirometry-IOS Digital System (Jaeger Company, Würtzburg, Germany) by the same experienced operator for all children and according to the American Thoracic Society’s recommendations [21]. The children breathed normally into a mouthpiece for a short period of time during which a loudspeaker delivered a pulse-shaped pressure flow excitation response to their respiratory systems. Parameters included the following: 1) resistance at oscillation frequencies of 5 Hz (R5, in kPa/L/s) and 20 Hz (R20, in kPa/L/s), 2) frequency dependence of resistance calculated as the change in resistance between 5 and 20 Hz (R5-R20, in kPa/L/s), 3) reactance at the oscillation frequency of 5 Hz (X5, in kPa/L/s), and 4) reactance area (AX, in kPa/L). Respiratory resistance at R5 and R20 were used as indices of total and proximal airway resistance, respectively. The fall in resistance from 5 Hz to 20 Hz (R5-R20, in kPa/L/s) was considered to be an index of the resistance of the peripheral airways [26, 27]. Moreover, reactance at 5 Hz (X5, in kPa/L/s) and the integral of the area of low-frequency reactance (AX, in kPa/L) were considered representative markers of peripheral airway abnormalities [28–30]. The bronchodilator response (BDR) was assessed by repeating the measurements 15 min after the inhalation of 200 μg of salbutamol and was calculated as the absolute change and relative change (percentage change from baseline) in IOS variables.

### Statistics

All statistical analyses were performed using SPSS software 23.0 (SPSS Inc., Chicago, IL, USA). Variables are expressed as the mean ± standard deviation unless otherwise specified. The *t*-test was used to compare continuous variables between the 2 groups and One-way ANOVA was used to compare continuous variable between the 3 groups. The correlations between IOS and airborne level during sleep were analyzed by Pearson’s correlation test. Fisher’s exact test was conducted to compare categorical variables. Figures were generated using GraphPad Prism 5.0 (Graphpad, San Diego, CA, USA). A p-value less than 0.05 was considered statistically significant.

## Results

### Clinical characteristics of the study subjects

There were 24 children with no underlying disease who were only exposed to a mixture of CMIT/MIT. Table 1 illustrates the general characteristics of the subjects. The mean age at the initial exposure to HD was 15.8 months. A third of the subjects reported exposure from the fetal period including one subject who was exposed only during the fetal period. Girls were predominant and were represented more than twice as often as boys were. The age distribution at the assessment of IOS varied from 3 to 12 years, but most of the children (9/15, 60%) were under 8 years of age. More children intermittently used a humidifier only during the winter season (62.5%) than those who intensively used it regardless of the season (37.5%). The mean period of HD use was 14.7 months. According to the diagnostic criteria, there were two definite, two probable, eight possible, and 12 unlikely cases. Hence, 16.7% (4/24) of the study subjects were determined to have definite or probable cases. Among the children who were only exposed to a mixture of CMIT/MIT without an underlying disease, death was occurred in 4.2% (1/24).

**Table 1:** General characteristics of study subjects.

		Frequency or mean	% or SD
**Age at initial exposure (months)**		15.8	23.4
**Fetal exposure**		8/24 (only used prenatally: 1/8)	33.3
**Sex (male: female)**		7:17	29.2:70.8
**Intense: intermittent use**		9:15	37.5:62.5
**Total months of use**		14.7	12.7
**Diagnostic** **criteria**	**Definite**	2	8.3
	**Probable**	2	8.3
	**Possible**	8	33.3
	**Unlikely**	12	50.0
**Death**		1	4.2

SD, standard deviation.

Table 2 demonstrates the case series. The definite and probable cases have a common feature in the HD exposure index. All children who were exposed initially as infants. The use of HD was intense and the duration was found to be less than 6 months in all cases. Among them, case number 10 had no previous disease history before or after birth. However, the subject died at the age of 4 months after continuous use of HD over 3 months right after birth.

**Table 2:** List of registered children with exposure to a mixture of CMIT and MIT.

Round	Case	Age at initial exposure (m)	Sex	Intense or intermittent use	Total use	Age at IOS (y)	Diagnostic criteria
**1**	**1**	31	M	Intense	1 m	NA	Unlikely
	**2**	25	F	Intense	10 m	NA	Unlikely
	**3**	13	F	Intermittent	3 y	NA	Unlikely
	**4**	45	M	Intermittent	2 y	NA	Unlikely
	**5**	83	F	Intermittent	5 y	NA	Unlikely
**2**	**6**	3	F	Intense	3 m	NA	Definite
	**7**	3	F	Intense	3 m	3.1	Definite
	**8**	Fetus	M	Intermittent	3 y	NA	Unlikely
	**9**	Fetus	F	Intense	18 m	NA	Unlikely
**3**	**10**	0	F	Intense	3 m	NA	Probable
	**11**	Fetus	F	Intermittent	3 y	6.9	Possible
	**12**	0	F	Intermittent	2 y	6.3	Possible
	**13**	Fetus	M	Intermittent	3 y	6.8	Possible
	**14**	Fetus	F	Intermittent	2 y	6.6	Possible
	**15**	5	F	Intermittent	3 y	7.9	Possible
	**16**	Fetus	F	Intermittent	2 y	9.3	Possible
	**17**	64	M	Intermittent	2 y	11.7	Possible
	**18**	8	F	Intense	6 m	11.2	Probable
	**19**	34	M	Intense	23 m	12.4	Possible
	**20**	Only as a fetus	M	Intense	8 m	4.7	Unlikely
	**21**	9	F	Intermittent	2 y	7.4	Unlikely
	**22**	0	F	Intermittent	3 y	7.5	Unlikely
	**23**	Fetus	F	Intermittent	2 y	9.4	Unlikely
	**24**	41	F	Intermittent	2 y	10.0	Unlikely

m, months; y, years; M, male; F, female; IOS, impulse oscillometry; NA, not available.

### Relationship between diagnostic criteria and HD exposure characteristics

Comparing the exposure index between definite/probable cases (n = 4) and unlikely cases (n = 12) in Table 3, the mean airborne disinfectant exposure intensity showed no statistical difference with a value of 10.9 μg/m^3^ among definite/probable cases and 10.4 μg/m^3^ among unlikely cases. However, mean airborne disinfectant exposure intensity during sleep among the definite/probable cases (32.4 μg/m^3^) was approximately twice that of those who had unlikely cases (17.3 μg/m^3^), showing a statistically significant difference (p = 0.026). As all the subjects in both groups were exposed during sleep, the difference in CMIT/MIT airborne disinfectant exposure intensity during sleep is believed to be significant. The age at the initial exposure was 3.5 months in the definite/probable cases. This is a much younger age than the 22.5 months in the unlikely cases, showing a statistically significant difference (p = 0.039). All of the definite/probable cases were intensive-use children, compared with only 33.3% of the unlikely cases. However, differences in the distance from the humidifier to the sleeping place and the direction of dispersal into the room showed no statistical significance. We also provided additional comparison including possible cases to the S1 Table.

**Table 3:** A comparison of exposure characteristics according to diagnostic criteria (Definite/Probable vs. Unlikely).

Exposure index		Definite/Probable (n = 4)	Unlikely (n = 12)	p-value
**Airborne disinfectant** **exposure intensity (**μ**g/m**^**3**^**)**		10.9 (± 2.6)	10.4 (± 6.5)	0.888
**Airborne disinfectant exposure intensity during sleep (**μ**g/m**^**3**^**)**		32.4 (± 8.7)	17.3 (± 11.0)	0.026^a^
**Use of HD during sleep (%)**		100%	100%	NA
**Total months of use**		3.8 (± 1.5)	17.6 (± 16.3)	0.120
**Age at initial exposure (months)**		3.5 (± 3.3)	22.5 (± 26.2)	0.039^a^
**Intense or intermittent use (%)**		100/0	33.3/66.7	0.077
**Distance** **between the bed and humidifier (%)**	**D < 0.5 m**	0	16.7	NA
	**0.5 m ≤ D < 1 m**	0	16.7	
	**1 m ≤ D < 2 m**	75	58.3	
	**2 m ≤ D**	25	8.3	
**Direction dispersed into room (%)**	**Forward**	50	63.6	1.000
	**Diagonal**	50	36.4	

HD, humidifier disinfectants; NA, not available; m, meter; D, distance.

^a^ p < 0.05 depicts a statistically significant difference.

### IOS parameters for exposure to a mixture of CMIT/MIT

Table 4 illustrates oscillometry data after categorizing subjects based on airborne disinfectant exposure intensity during sleep. For high-density exposure subjects (exposure to a level above the mean among the definite/probable cases was defined as high density [≥ 32.4 μg/m^3^]), IOS measurements demonstrated peripheral airway involvement, specifically airway reactance, compared to low-density exposure (exposure to less than that level was defined as low density [< 32.4 μg/m^3^]). The X5 was −0.463 kPa/L/s in the high-density exposure group. This is a more negative value compared to the low-density exposure group at −0.296 kPa/L/s, showing a statistically significant difference (p = 0.001). Moreover, the high-density exposure group also showed an AX of 3.240 kPa/L, which was a greater increase than that seen in the low-density exposure group at 1.922 kPa/L (p = 0.039). Additionally, we have investigated the association between IOS data and diagnostic criteria (S2 Table). The tendency is similar to comparison of the airborne disinfectant exposure intensity during sleep indicating a more decreased X5, increased AX, and increased R5-R20 in Definite/Probable children.

**Table 4:** Impulse oscillometry parameters for high-density exposure and low-density exposure during sleep.

		High-density exposure (n = 3)	Low-density exposure (n = 12)	p-value
**Height (cm)**		119.0	125.0	0.233
**Weight (kg)**		22.0	25.5	0.295
**BMI (kg/m**^**2**^**)**		16.0	16.0	0.945
**IOS**	**R5, kPa/L/s**	1.1533	0.9092	0.106
	**R20, kPa/L/s**	0.8067	0.6783	0.192
	**R5-R20, kPa/L/s**	0.3467	0.2308	0.078
	**X5, kPa/L/s**	-0.4633	-0.2958	0.001^a^
	**AX, kPa/L**	3.2400	1.9217	0.039^a^

High-density exposure = airborne level during sleep ≥ 32.4 μg/m^3^; low-density exposure = airborne level during sleep < 32.4 μg/m^3^. R5, resistance at 5 Hz; R20, resistance at 20 Hz; R5-R20, change in resistance between 5 and 20 Hz; X5, reactance at 5 Hz; AX, reactance area.

^a^ p < 0.05 depicts a statistically significant difference.

No significant difference was seen in airway resistance at R5 (an index of total airway resistance) or R20 (an index of proximal airway resistance). However, there was variance among the groups (p = 0.078) in the weak difference between R5 and R20 (= R5-R20, an index of peripheral airway resistance), which suggested that the airway obstruction might only demonstrate peripheral airway dysfunction.

**Fig 1:**
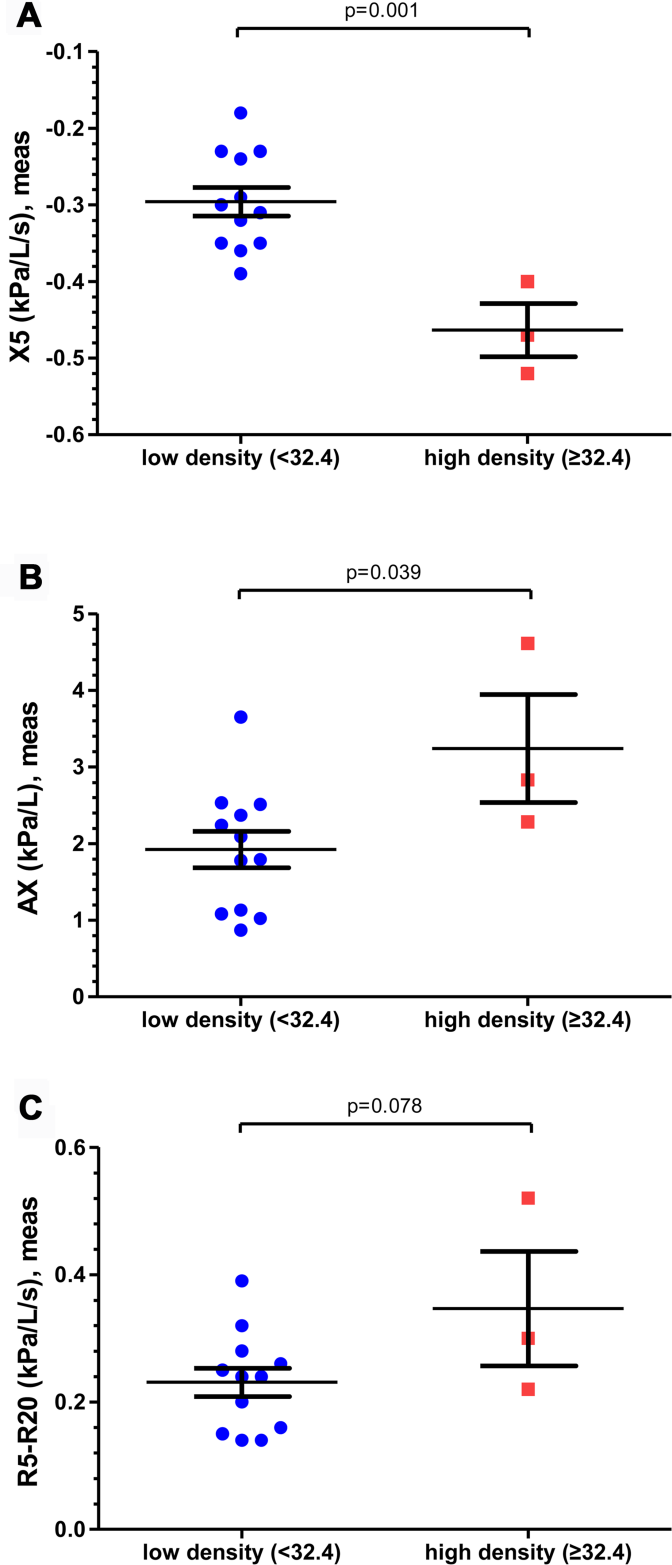
Comparisons of the mean values for IOS parameters according to the airborne level during sleep. X5 (A), AX (B), R5-R20 (C), and error bars indicate standard errors of the means.

**Fig 2:**
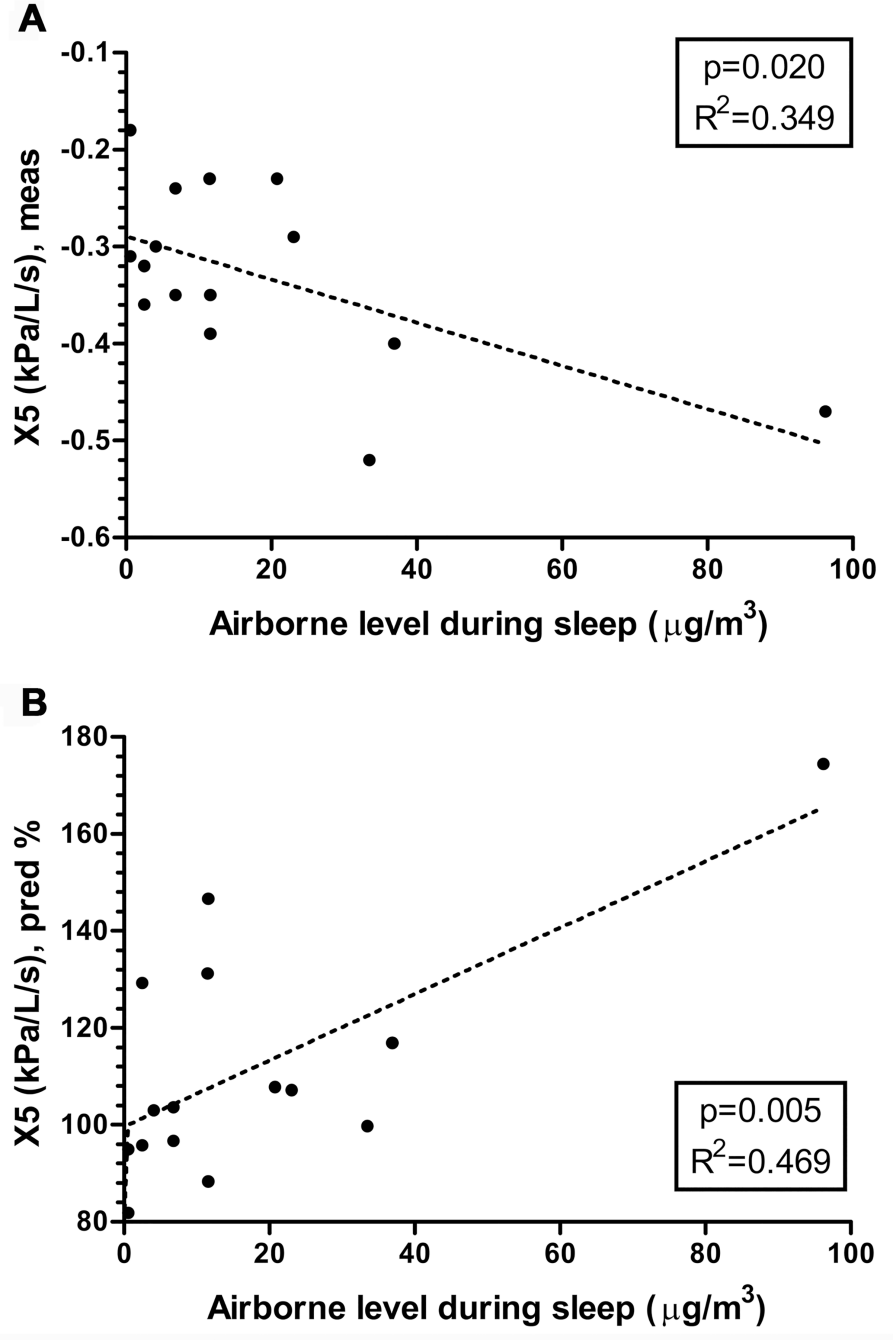
Correlation between airborne level during sleep and X5. Measured in kPa/L/s (A) or predicted percentage (B).

As shown in Fig 1, R5-R20, X5, and AX, which are specific to the peripheral airway, showed differences according to airborne disinfectant exposure intensity during sleep. Data were not different (p > 0.05) between the groups in height, weight, or BMI. Based on these results, relationships between X5 and airborne level during sleep were analyzed for exposure to a mixture of CMIT/MIT in Fig 2. X5 (measured in kPa/L/s) was inversely correlated with airborne level during sleep (R^2^ = 0.349; p = 0.020) and the X5 predicted percentage was directly correlated with airborne level during sleep (R^2^ = 0.469; p = 0.005).

In addition, X5 decreased more negatively among the highest tertile compared to the middle and lowest tertiles (p = 0.007, S1 Table and S1 Fig).

### Irreversibility in reactance

**Fig 3:**
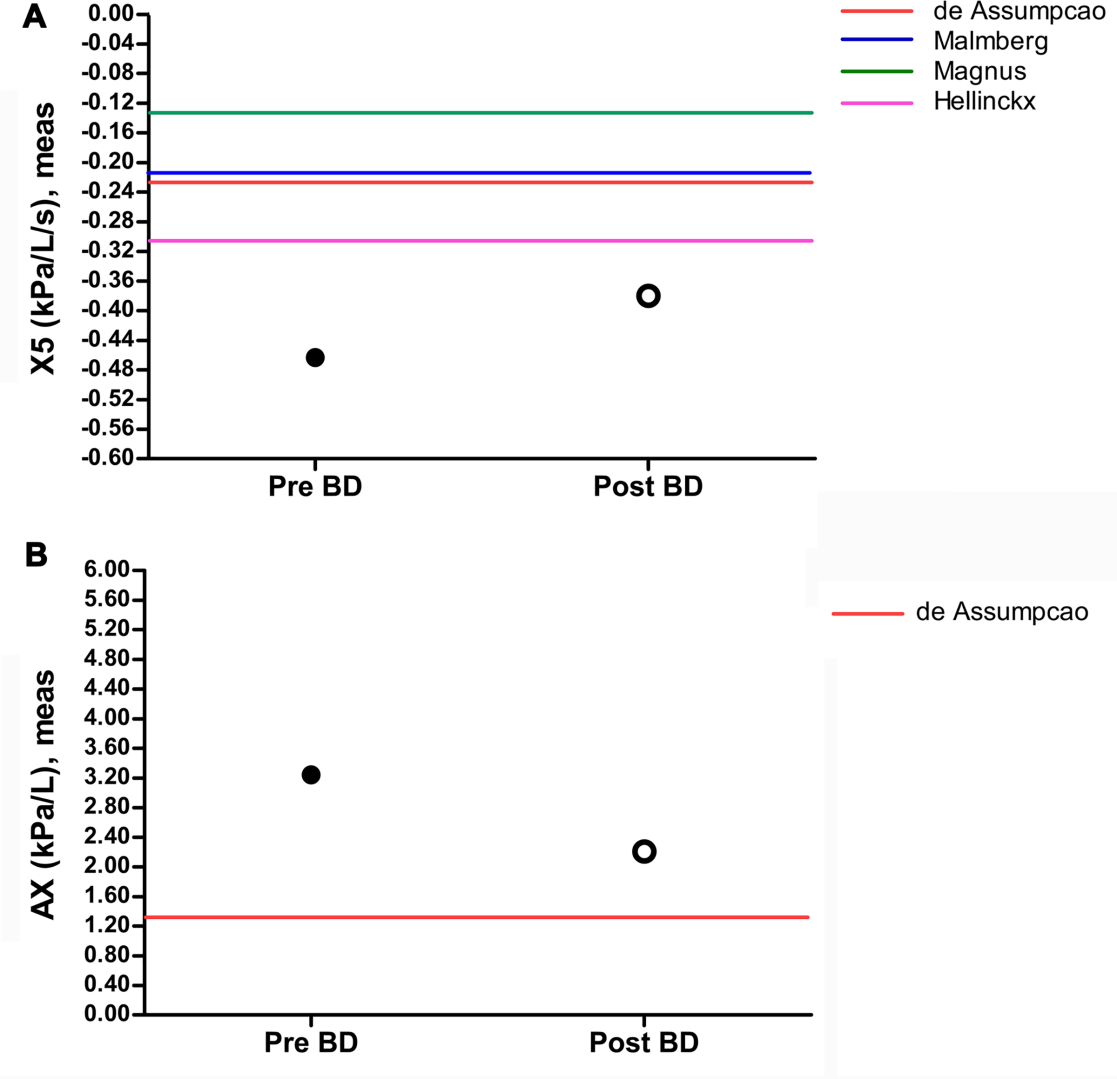
Bronchodilator response for IOS parameters (X5 and AX) in children with high-density exposure during sleep. Mean values are plotted before and after bronchodilator administration. The line represents the published normal for each parameter.

Fig 3 illustrates response to a bronchodilator in children with high-density exposure during sleep in baseline reactance data for X5 and AX, indicating the peripheral airway.. Although values changed following bronchodilator administration, the magnitude of the change was relatively small and persistent abnormalities were noted in children with high-density exposure during sleep (X5, -0.380 kPa/L/s vs. baseline [p = 0.167]; AX, 2.210 kPa/L vs. baseline [p = 0.462]), and AX was −43.567 kPa/L (p = 0.167 compared to the published normal reference value) [31–34]. These findings provide insight into the pathologic mechanism of peripheral airway dysfunction due to bronchiolar destruction and the centrilobular distribution of alveolar destruction following HD exposure (Lee et al. 2013), indicating irreversible change in the present study.

## Discussion

This study demonstrates peripheral airway dysfunction as assessed by IOS following only exposure to a mixture of CMIT/MIT. The abnormality showed a more decreased X5 and increased AX in reactance, with non-response to a bronchodilator. Thus, these data suggested peripheral airway dysfunction, confirming irreversibility. Although the majority of the patients are young children and spirometry was not available, irreversible peripheral airway dysfunction assessed by IOS in our study demonstrated a possibility of inhalation toxicity that was expected to be considerably severe, resulting in pulmonary damage. This evidence may help clinicians, patients, families, and other workers to better characterize the role of a mixture of CMIT/MIT in the respiratory system. And IOS parameters provide a new biomarker for the assessment of lung function in young children with HDLI, which in many cases may be difficult to evaluate.

To date, only a limited number of studies have provided IOS parameters of adult populations with environmental exposure that resulted in restrictive lung function, including the World Trade Center disaster [35]. To the best of our knowledge, this is the first study to reveal irreversible peripheral airway dysfunction from exposure to a mixture of CMIT/MIT in humans and the first pediatric study finding an association between environmental chemical exposure and pulmonary function using IOS.

Compared with contact dermatitis from a mixture of CMIT/MIT, only few case have been reported in which a mixture of CMIT/MIT was inhaled originating from water-based paint, which was suggested to provoke respiratory disease such as occupational asthma in humans [3]. In animal studies, the inhalation of a mixture of CMIT/MIT has been shown to cause decreased weight gain and pulmonary hemorrhages in rats [4]. Another study also reported cellular toxicity to rat alveolar macrophages from inhalation [20]. This study suggested that CMIT markedly inhibited phagocytic oxidative burst and cellular cytokine (Tumor necrosis factor-alpha, TNF-α) secretion. That is, CMIT acted by inhibiting cellular functions that governed oxidative burst and hence the release of all reactive oxygen species, as well as inhibiting the secretion of TNF-α. These findings provide some corroboration that exposure to this chemical may cause inhalation injury including HDLI in Korea. Thus, the potential respiratory health risks of a mixture of CMIT/MIT inhalation must be investigated, and this is the first study to reveal adverse respiratory effects, especially involving the peripheral airway, from exposure to a mixture of CMIT/MIT in children.

Interestingly, airborne disinfectant exposure density during sleep was more important than the usual airborne exposure density in causing HDLI in our study. Several hypotheses may explain the possibilities in this situation. Most respondents reported using HD during sleep in a room with little ventilation because a young child’s room is a relatively confined space, and generally, parents tend to close the window when their children sleep. Moreover, children, especially in infancy, cannot remove themselves from exposure during sleep. This can result in considerable cumulative exposure during sleep, even though only a small amount is used each time. Additionally, it is colder at night than during the day, and parents increase the room temperature. This would lead to greater humidifier use than usual because of the dry air.

Infants and young children are suspected to be at high risk since they take in more of the contaminant, such as a disinfectant, than adults relative to their body size, and have particularly vulnerable physiologies [16]. Infants may be even more vulnerable than adults to these exposures, as the minute ventilation per pound in an infant is significantly more than that of an adult [36]. Children’s groups were found to have used HD intensively during a specific period of child development, such as in the pre-gestational, gestational, and post-natal periods. These periods may have caused increased susceptibility to lung injury.

Many occupational and environmental bronchiolar disorders describe varied impairment in lung function including obstructive, restrictive, and mixed patterns for each bronchiolar disorder [37–41]. However, adult cases of all HDLI, regardless of the strand of HD, had only a restrictive pattern on spirometry [13, 42]. Restrictive lung diseases will result in a decrease in reactance because the elastic recoil, and therefore the ability of the lung parenchyma to reflect the signal back, will be decreased [29]. This study also describes a restrictive lung function indicating peripheral airway dysfunction in reactance from specific strand “a mixture of CMIT/MIT” exposure in children. Notably, peripheral airway dysfunction in X5 and AX was not related to bronchodilator response in the present study. These results are compatible with prior publications’ results on HDLI addressing small airway involvement on computed tomography (CT) and a restrictive pattern on spirometry in adults [13, 42]. In addition, a previous study showed that bronchiolar destruction and a centrilobular distribution of alveolar destruction following HD exposure were documented by lung biopsy [9]. These imaging and pathologic findings may also explain the small magnitude of change and persistent abnormalities in IOS parameters (X5, AX) noted after the use of a bronchodilator.

An advantage of IOS may be the detection of subtle changes in a patient’s airway function earlier than with conventional spirometry [35, 43] and some data suggest that IOS can be used to assess abnormal distal airway function, even in the setting of normal spirometry [35, 44]. Especially, airway reactance was also correlated with ground-glass opacity [45].

IOS detected significant bronchodilator responses in areas of resistance and were nearly significant in reactance within an asthmatic group [46, 47]. Thus, the response to a bronchodilator in this study is considered to be a crucial finding to distinguish the disease from asthma.

In the present study, the reason why a comparison of pulmonary function according to exposure concentration is not a diagnostic criterion is as described below. First, many patients were reported to show a resolution of symptoms, despite the disease severity and very high prevalence of hypoxemia, even if they were hospitalized in an intensive care unit (ICU) in the past because of the passage of more than 5 years from the incident point. In particular, imaging findings in children who were exposed to a mixture of CMIT and MIT are currently almost normal (84.2%, 16/19) and no child underwent a pathologic biopsy. Cohort follow-up, especially related to pulmonary function test performance, will be important [48]. Therefore, since most of the differences in diagnostic criteria are dependent on lung function, lung function seems to be an alternative biomarker to establish diagnostic criteria. Consequently, it is important to compare the exposure concentration with lung function directly. Second, the patients who were exposed to a mixture of CMIT and MIT were a small population overall compared with other HD strands and most patients diagnosed with HDLI were not able to perform a pulmonary function evaluation because of their younger age, inability (e.g., death), or lack of cooperation. However, we were successful in calculating all patients’ exposure characteristics. Therefore, we simply investigated significant changes in lung function in accordance with exposure to a mixture of CMIT and MIT as an objective indicator. Hereafter, a greater number of patients who were exposed to a mixture of CMIT and MIT needs to be studied to further strengthen our results, and investigate but also other effective biomarkers directed at specific pathways in specific individuals, and not only pulmonary function.

Although promising results were obtained, we are aware that this study has limitations. First, the number of patients included in the study was relatively small. To verify the results, a study with a larger sample size is necessary. Second, we could not explore the follow-up data, and the long-term clinical significance with respect to lung function is still unknown. Follow-up is needed for a longer duration, and we should investigate the trajectory of lung function in those exposed to a mixture of CMIT/MIT in the near future. Third, there could be a risk of recall bias, because most disinfectant use characteristics were obtained predominantly from a direct questionnaire given to the study subjects. We concluded that there might be a small possibility of confusion regarding the only HD brand they used because we selected subjects who used only HD brands containing a mixture of CMIT and MIT. Even though there could be a recall bias about the duration of HD use, it was not hard for them to recall the type, color, and size of product that can be specific to the brand or type of HD. We increased the accuracy of responses by showing a photograph of each disinfectant brand and asking additional questions that may be specific to the respective disinfectant types.

## Conclusions

This study shows that peripheral airway dysfunction in children was associated with high-density exposure during sleep to a mixture of CMIT and MIT, including decreased X5 and increased AX in reactance, with no responsive to a bronchodilator. The results of our study confirm significant damage to the small airways in the pathophysiology of exposure to a mixture of CMIT and MIT. Furthermore, IOS provides a new biomarker for the assessment of lung function in young children with HDLI.

## Supporting information

S1 Table A comparison of exposure characteristics according to diagnostic criteria (Definite/Probable vs. Possible vs. Unlikely). HD, humidifier disinfectants; NA, not available; m, meter; D, distance. ^a^ p < 0.05 depicts a statistically significant difference over several groups.
(DOCX)

S2 Table Impulse oscillometry parameters for diagnostic criteria. R5, resistance at 5 Hz; R20, resistance at 20 Hz; R5-R20, change in resistance between 5 and 20 Hz; X5, reactance at 5 Hz; AX, reactance area. ^a^ p < 0.05 depicts a statistically significant difference over several groups.
(DOCX)

S3 Table Impulse oscillometry parameters according to the tertile of exposure density during sleep. ^a^ p < 0.05 depicts a statistically significant difference over several groups.
(DOCX)

S1 Fig X5 parameters according to the tertile of exposure density during sleep. (TIF)
