# Supplementary material for: Effects of a mixture of chloromethylisothiazolinone and methylisothiazolinone on peripheral airway dysfunction in children
Source: PLoS One. 2017 Apr 28;12(4):e0176083. doi: 10.1371/journal.pone.0176083 (PMC5409534; doi:10.1371/journal.pone.0176083)
Supplement: S1 Table — HD, humidifier disinfectants; NA, not available; m, meter; D, distance. a p < 0.05 depicts a statistically significant difference over several groups. (DOCX) [file pone.0176083.s001.docx]

**S1 Table. A comparison of exposure characteristics according to diagnostic criteria (Definite/Probable vs. Possible vs. Unlikely)**

| Exposure index | | Definite/  Probable  (n = 4) | Possible  (n=8) | Unlikely  (n = 12) | p-value |
| --- | --- | --- | --- | --- | --- |
| Airborne disinfectant  exposure intensity (µg/m^3^) | | 10.9 (± 2.6) | 12.7 (± 21.7) | 10.4 (± 6.5) | 0.928 (One-way ANOVA)  0.232 (Kruskall Wallis) |
| Airborne disinfectant exposure intensity during sleep (µg/m^3^) | | 32.4 (± 8.7) | 18.6 (± 32.3) | 17.3 (± 11.0) | 0.439 (One-way ANOVA)  0.038^a^ (Kruskall Wallis) |
| Use of HD during sleep (%) | | 100 | 100 | 100 | NA |
| Total months of use | | 3.8 (± 1.5) | 15.8(± 5.1) | 17.6 (± 16.3) | 0.162 (One-way ANOVA)  0.017^a^ (Kruskall Wallis) |
| Age at initial exposure (months) | | 3.5 (± 3.3) | 12.9 (± 23.8) | 22.5 (± 26.2) | 0.363 (One-way ANOVA)  0.435 (Kruskall Wallis) |
| Intense or intermittent use (%) | | 100/0 | 25.0/75.0 | 33.3/66.7 | 0.032^a^ |
| Distance between the bed  and humidifier (%) | D < 0.5 m | 0 | 0 | 16.7 | 0.163 |
|  | 0.5 m ≤ D < 1 m | 0 | 12.5 | 16.7 |  |
|  | 1 m ≤ distance < 2 m | 75 | 25.0 | 58.3 |  |
|  | 2 m ≤ distance | 25 | 62.5 | 8.3 |  |
| Direction dispersed into room (%) | Forward | 50 | 37.5 | 63.6 | 0.528 |
|  | Diagonal | 50 | 62.5 | 36.4 |  |

HD, humidifier disinfectants; NA, not available; m, meter; D, distance. ^a^ p < 0.05 depicts a statistically significant difference over several groups.
