# Supplementary material for: Effects of a mixture of chloromethylisothiazolinone and methylisothiazolinone on peripheral airway dysfunction in children
Source: PLoS One. 2017 Apr 28;12(4):e0176083. doi: 10.1371/journal.pone.0176083 (PMC5409534; doi:10.1371/journal.pone.0176083)
Supplement: S2 Table — R5, resistance at 5 Hz; R20, resistance at 20 Hz; R5-R20, change in resistance between 5 and 20 Hz; X5, reactance at 5 Hz; AX, reactance area. a p < 0.05 depicts a statistically significant difference over several groups. (DOCX) [file pone.0176083.s002.docx]

**S2 Table. Impulse oscillometry parameters for diagnostic criteria**

|  | | Definite/Probable  (n=2) | Possible  (n=8) | Unlikely  (n=5) | p-value |
| --- | --- | --- | --- | --- | --- |
| Height (cm) | | 115.5 | 128.1 | 122.5 | 0.649 |
| Weight (kg) | | 24.0 | 27.8 | 26.5 | 0.921 |
| BMI (kg/m^2^) | | 16.8 | 16.1 | 17.1 | 0.697 |
| IOS | R5, kPa/L/s | 1.0200 | 0.8713 | 1.0720 | 0.315 |
|  | R20, kPa/L/s | 0.6850 | 0.6437 | 0.8080 | 0.152 |
|  | R5-R20, kPa/L/s | 0.3350 | 0.2275 | 0.2640 | 0.430 |
|  | X5, kPa/L/s | -0.3750 | -0.3188 | -0.3280 | 0.771 |
|  | AX, kPa/L | 2.8450 | 1.8912 | 2.3920 | 0.456 |

R5, resistance at 5 Hz; R20, resistance at 20 Hz; R5-R20, change in resistance between 5 and 20 Hz; X5, reactance at 5 Hz; AX, reactance area. ^a^ p < 0.05 depicts a statistically significant difference over several groups.
