# Supplementary material for: Effects of a mixture of chloromethylisothiazolinone and methylisothiazolinone on peripheral airway dysfunction in children
Source: PLoS One. 2017 Apr 28;12(4):e0176083. doi: 10.1371/journal.pone.0176083 (PMC5409534; doi:10.1371/journal.pone.0176083)
Supplement: S3 Table — a p < 0.05 depicts a statistically significant difference over several groups. (DOCX) [file pone.0176083.s003.docx]

**S3 Table. Impulse oscillometry parameters according to the tertile of exposure density during sleep**

|  | | Highest tertile  (n = 3) | Middle tertile  (n = 5) | Lowest tertile  (n = 7) | p-value |
| --- | --- | --- | --- | --- | --- |
| Height (cm) | | 119.0 | 132.0 | 120.0 | 0.422 |
| Weight (kg) | | 22.0 | 28.0 | 23.0 | 0.527 |
| BMI (kg/m^2^) | | 16.0 | 16.1 | 16.0 | 0.851 |
| IOS | R5, kPa/L/s | 1.1533 | 0.8840 | 0.9271 | 0.271 |
|  | R20, kPa/L/s | 0.8067 | 0.6780 | 0.6786 | 0.442 |
|  | R5-R20, kPa/L/s | 0.3467 | 0.2060 | 0.2486 | 0.170 |
|  | X5, kPa/L/s | -0.4633 | -0.2980 | -0.2943 | 0.007^a^ |
|  | AX, kPa/L | 3.2400 | 1.7780 | 2.0243 | 0.117 |

^a^p < 0.05 depicts a statistically significant difference over several groups.
