# Supplementary figures and images for: Effects of a mixture of chloromethylisothiazolinone and methylisothiazolinone on peripheral airway dysfunction in children
Source: PLoS One. 2017 Apr 28;12(4):e0176083. doi: 10.1371/journal.pone.0176083 (PMC5409534; doi:10.1371/journal.pone.0176083)

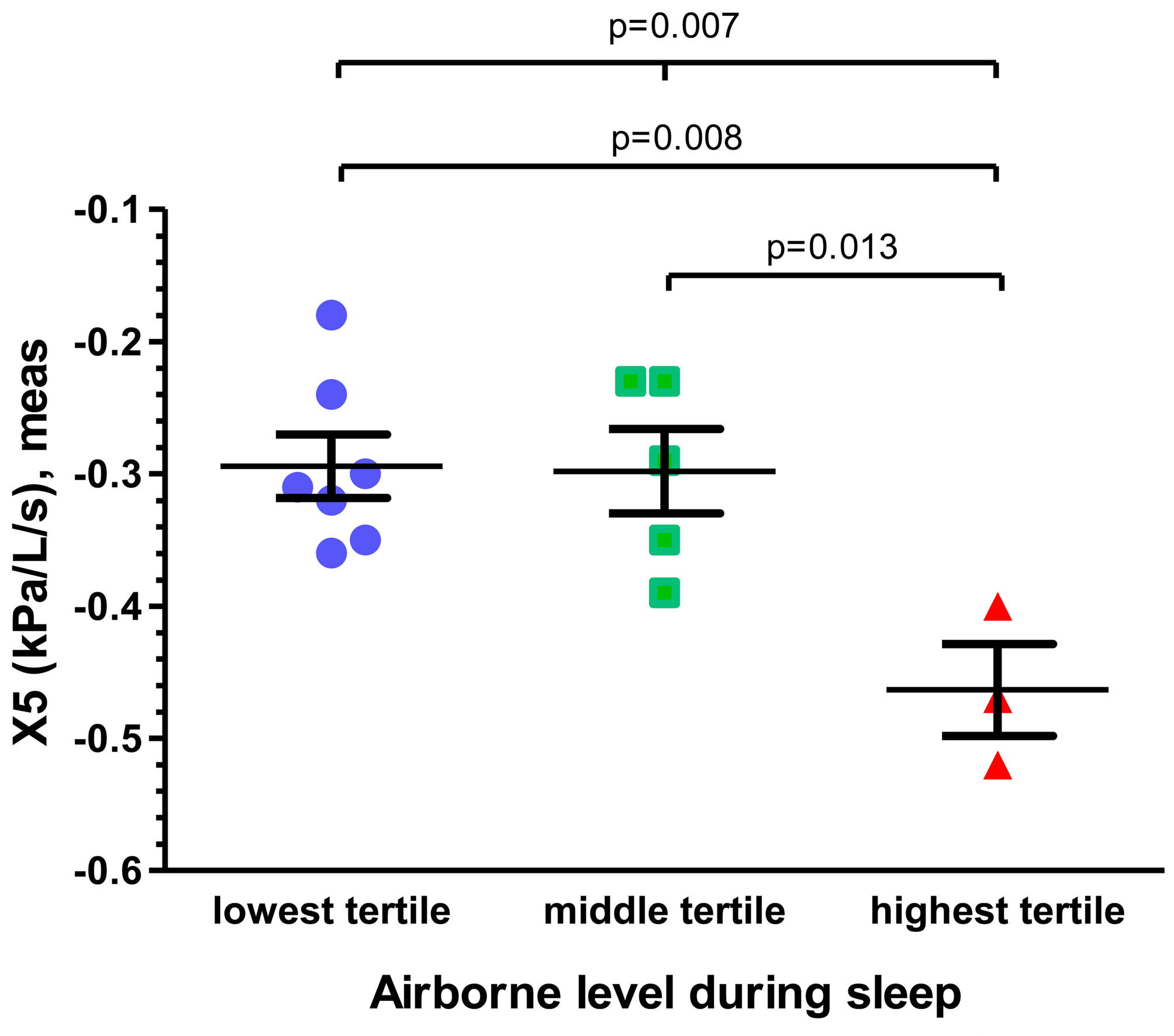

Supplement: S1 Fig — (TIF) [file pone.0176083.s004.tif]
